# *Leptospira *seroprevalence and associations between seropositivity, clinical disease and host factors in horses

**DOI:** 10.1186/1751-0147-51-15

**Published:** 2009-03-30

**Authors:** V Båverud, A Gunnarsson, E Olsson Engvall, P Franzén, A Egenvall

**Affiliations:** 1 Department of Bacteriology, National Veterinary Institute, SE-751 89 Uppsala, Sweden; 2 Department of Clinical Sciences, Faculty of Veterinary Medicine, Swedish University of Agricultural Sciences, P.O. Box 7019, SE-750 07 Uppsala, Sweden; 3 Section of Ruminant Medicine and Veterinary Epidemiology, Faculty of Veterinary Medicine, Swedish University of Agricultural Sciences, P.O. Box 7019, SE-750 07 Uppsala, Sweden

## Abstract

**Background:**

A cross-sectional study was carried out to determine the seroprevalence of different serovars of *Leptospira *spp. and their association with clinical disease and host factors in Swedish horses.

**Methods:**

Sera from 2017 horses brought to equine clinics during 1997–98 were investigated. The sera were examined by microscopic agglutination test for the presence of antibodies against the following *L. interrogans *serovars: Bratislava strain Jez, Icterohaemorrhagiae strain Kantorowicz and Pomona strain Pomona and also *L. kirschneri *sv Grippotyphosa strain Duyster and *L. borgpetersenii *sv Sejroe strain M 84. Host factors, disease factors, season, pasture access and outdoor confinement variables were analysed with respect to seropositivity to sv Bratislava and Icterohaemorrhagiae. Multivariable logistic regression was used to model seropositivity to sv Bratislava and Icterohaemorrhagiae (seroprevalence > 8%).

**Results:**

The seroprevalence, at a cut-off 1:100, were for sv Bratislava (16.6%), Icterohaemorrhagiae (8.3%), Sejroe (1.2%), Pomona (0.5%) and Grippotyphosa (0.4%). In the multivariable analysis, it was demonstrated that seroprevalence increased with age for sv Bratislava and Icterohaemorrhagiae. For sv Bratislava the seasons April – June and October – December and for sv Icterohaemorrhagiae October – December had higher seroprevalences than other seasons. Horses not used for racing had higher levels of seropositivity to sv Bratislava. Furthermore, horses with respiratory problems as well as horses with fatigue had higher levels of seropositivity to sv Bratislava. Ponies and coldbloods, and horses with access to pasture, had lower seroprevalence for sv Icterohaemorrhagiae. Healthy horses had lower seroprevalence for sv Icterohaemorrhagiae, than non-healthy horses.

**Conclusion:**

There was no significant association between clinical signs and disease and positive titres to sv Bratislava (except for the association between respiratory problems and fatigue and seropositivity to sv Bratislava). The results suggest that horses with increasing age and exposed to factors associated with outdoor life had an increased seroprevalence for sv Bratislava, indicating that horses get infected from outdoor and/or are exposed to shedding from other horses (management dependent). For sv Icterohaemorrhagiae, management possibly plays a role as ponies and coldbloods as well as healthy horses had lower seroprevalence. Overall, the age of the horse should be taken into consideration when evaluating the titre as the average healthy horse has a higher titre than a young horse.

## Background

Leptospirosis is a serious worldwide, zoonotic infectious disease of humans, domestic animals and wildlife, caused by any of the pathogenic serovars (sv) within the genus *Leptospira *(*L*.). Infection usually results from direct transmission via contaminated urine or placental fluid, or indirectly from a contaminated environment [1]. Clinical manifestations of leptospirosis vary from acute to subacute and chronic infection. Severe illness with jaundice, haemoglobinuria, renal failure, meningitis and abortions occur in domestic animals. Subclinical forms are perhaps more common with chronically infected animals which can be carriers for years to life [2].

Clinical infections have sometimes been observed in horses [3]. The organism can cause uveitis [4-6], abortion [7,8], stillbirth [9], prematurely born foals [10], renal dysfunction [11] and hepatic dysfunction [12]. Signs that have been observed include haematuria [13], fever, jaundice, anorexia [14-16], and respiratory distress [16].

Serological testing has been the traditional way of diagnosing leptospirosis in the laboratory. Microscopic agglutination test (MAT) is the standard reference serological test and a four-fold or greater change in antibody titres in paired acute and convalescent sera is considered diagnostic as well as a single finding of a high titre together with clinical signs. Isolation of the spirochete is the ideal situation; however, it is a difficult, time-consuming task for specialized reference laboratories [2]. In recent years, polymerase chain reaction (PCR) specifically amplifying leptospiral DNA has been used for demonstration of the organism in tissue from prematurely born foals [10,17] and in the vitreous aqueous humor of horses affected by recurrent uveitis [4,6].

Serological evidence of leptospiral infection is common in horses. Predominant serovars reported are *L. interrogans *sv Pomona, *L. interrogans *sv Bratislava, *L. interrogans *sv Icterohaemorrhagiae and *L. kirschneri *sv Grippotyphosa [3,18-26]. In Sweden, although laboratory confirmation by MAT, with a titre of ≥ 1:100, of any *Leptospira *serovar in horses is notifiable [27], there is limited knowledge of *Leptospira *seroprevalence in Swedish horses. Sometimes, mature horses have been brought to veterinarians due to vague clinical signs, e.g. poor performance or intermittent fever and serological testing for antibodies to *Leptospira *has been performed. Positive results have due to the limited knowledge sometimes been difficult to interpret, which prompted us to perform this investigation.

The aims of the study were to estimate the seroprevalence of several *Leptospira *spp. in Swedish healthy and non-healthy horses brought to equine clinics and to investigate possible associations of seropositivity of the organism with clinical disease status and host factors.

## Materials and methods

### Sampling procedure

A cross-sectional study was performed from 1 September 1997 to 3 September 1998 to determine the seroprevalence of five different serovars of *Leptospira *in horses. The same material was used previously for an investigation of seropositivity to *Borrelia burgdorferi *sensu lato and granulocytic *Ehrlichia *spp. (*Anaplasma phagocytophilum*) [28]. Blood samples were collected from horses brought to major equine clinics (*n *= 17) affiliated to the Swedish Horserace Totalizator Board. Each clinic was assigned at random (by lottery) 2 days in each month when they sampled horses up to a predetermined quota (3–19 each month). The quota was directly proportional to the number of cases examined at these clinics during the previous year. The purpose was to sample approximately 2000 horses. Sampling calculations were performed mainly for the previous study [28].

All owners/trainers were informed and invited to participate. They completed a questionnaire regarding both the horses' health status and host factors [28]. The clinic veterinarians also answered questions about the horses' health, most closed and semi-open, except for one open question concerning the diagnosis. For this study, an additional question was included in the questionnaire regarding the occurrence of rodents in the stables (none, few, many, not known). The questionnaires, together with the blood samples, were sent to the National Veterinary Institute, Uppsala, Sweden. Samples were centrifuged and the sera were stored at -20°C until tested. The study was approved by the Swedish Ethical Committee on Animal Experiments.

### Animals

A total of 2031 horses were sampled. Of these, samples from 14 horses were excluded (12 lacked most of the information required by the questionnaires and two lacked a serum sample). A total of 2017 horses of different breeds, age and sex were evaluated in the study (Table 1), grouped healthy (*n *= 400) and non-healthy horses (*n *= 1617). The purpose of the visit, with various alternatives such as disease, castration, pre-purchase examination, vaccination, lameness, teeth floating or other, and the clinical signs were recorded by the veterinarian.

**Table 1:** Distribution of seroprevalences for *Leptospira interrogans *serovars Bratislava and Icterohaemorrhagiae, cut-off 1:100, by breed, age, gender, usage, season, location, pasture access, outdoor confinement and occurrence of rodents in 2017 horses^a^

		% positive horses	
		
Variable	No. of tested horses	sv Bratislava	sv Icterohaemorrhagiae
Breed			
Standardbred	912	8.7	6.6
Thoroughbred	137	16.8	5.8
Pony	174	27.0	9.2
Coldblood	157	19.1	4.5
Warmblood	637	24.5	12.1
Age (years)^b^			
0–2	231	6.9	1.7
3–5	668	11.8	4.5
6–10	727	17.7	8.9
11–15	289	19.0	17.0
> 15	99	35.4	19.2
Gender			
Mare	807	20.8	8.4
Gelding	916	16.6	9.7
Stallion	294	5.1	3.7
Usage			
Trotting	964	8.2	6.0
Exercise	446	25.6	10.5
Competition riding	443	25.3	11.5
Riding school	106	24.5	15.1
Gallop	69	8.7	4.3
Breeding	57	29.8	7.0
Other	96	20.8	8.3
Season			
January-March	514	11.3	7.0
April-June	507	21.9	6.3
July-September	483	16.1	9.1
October-December	513	17.2	10.9
Region^c^			
South	824	18.2	9.1
Middle	588	16.8	8.5
North	572	14.9	7.3
Pasture access^d^			
None	598	11.9	9.4
< 1 month	175	20.6	5.1
1–3 months	530	19.4	10.9
> 3 months	488	19.7	5.3
Outdoor confinement			
None	23	8.7	0
Yard without grass	750	13.5	7.9
Field with grass	1248	17.9	9.0
Riding in terrain	369	21.1	9.5
Occurrence of rodents^e^			
None	387	19.6	23.0
Few	1443	15.7	20.9
Many	52	17.3	19.2
Not known	123	18.7	23.6

^a^Horses were sampled during September 1997–98 from clinics associated with the Swedish Horserace Totalizator Board. ^b^For three horses, information on age was missing. ^c^For 33 horses, information on location was missing.^d^For 226 healthy horses, information on pasture access was missing. ^e^For 12 horses, information on occurrence of rodents was missing.

### Healthy horses (*n *= 400)

This group comprised horses that were considered entirely fit for the task required and deemed healthy by the owner/trainer at the time of sampling and also during the previous 12 months. Some horses (*n *= 251) were brought to the clinics for procedures not associated with clinical disease such as vaccination, teeth floating, pre-purchase examination and castration. Horses were included in the group only if the veterinarian deemed the horse to be healthy. Other horses (*n *= 149) brought to the clinic only for acute traumatic injuries were included in the healthy group if they had been healthy during the previous year. It was assumed that such injuries were unlikely to be associated with *Leptospira *infection.

### Non-healthy horses (*n *= 1617)

The clinical signs, aetiology or procedures recorded were: abortion, behavioural problems, colic, diarrhea, fatigue, fever, inappetence, integumentary problems, lame 1 leg, lame ≥ 2 legs, ophthalmological disease, radiographic examination (admitted for various problems to the equine clinics by veterinarians in horse practice), respiratory signs, staggering, stiffness, tendon injuries, traumatic injuries, unwillingness to be ridden or wasting (all denoted as disease factors). Lameness was analysed for one or for several limbs as well as for "any lameness", irrespective of the number of lame limbs. The variable "tendon injuries" represents only cases with clinically confirmed tendon injuries. The variable "unwillingness to be ridden" was analysed within the group of riding horses (horses used for exercise, competition, or as riding school horses and of pony or warmblood breeds).

### Serological test to *Leptospira*

The 2017 sera were examined by MAT [2]. The antigens used were live cultures of reference strains *L. interrogans *sv Bratislava, strain Jez (sv Bratislava); *L. interrogans *sv Icterohaemorrhagiae, strain Kantorowicz (sv Icterohaemorrhagiae); *L. kirschneri *sv Grippotyphosa, strain Duyster (sv Grippotyphosa); *L. interrogans *sv Pomona, strain Pomona (sv Pomona); and *L. borgpetersenii *sv Sejroe, strain M 84 (sv Sejroe). These reference strains were obtained from the Royal Tropical Institute in Amsterdam, The Netherlands. The serovars tested for in this study represent a spectrum that was expected to be prevalent among horses in Sweden, according to an earlier study [19] and information gained by experience from testing of clinical samples at the diagnostic laboratory at the National Veterinary Institute, Uppsala.

All sera that gave a positive reaction at a 1:100 dilution were further titrated in serial two-fold dilutions to titre end-point, i.e. 50% agglutination [2]. A titre ≥ 1:100 was deemed positive, i.e. indicating exposure to leptospires.

#### Quality control

Purity of the antigens used in the MAT was checked regularly by microscopy. An evaluation of the identity of the antigens against reference sera obtained from the Royal Tropical Institute in Amsterdam, the Netherlands was performed twice a year. The MAT was performed by one person, to limit inter-observer variations of the test results.

### Data analysis (description of variables, variable construction and data analysis)

Horses were considered as 0 years old during the year born and one year the following year, etc. "Warmblood" included mostly Swedish Warmbloods, while "coldbloods" included horses such as North-Swedish horses, Icelandic horses, and Fiord horses. Owners/trainers categorized the usage of the horses into the following given categories: 1) trotting competition, 2) gallop racing, 3) competition riding (mainly dressage, show-jumping or 3-day-eventing), 4) exercise riding, 5) breeding or 6) other (open alternative). More than one option for usage could be marked. According to the open option, several horses were riding-school horses and this was therefore retained as a category. The owners/trainers also answered questions about outdoor confinement, pasture access, and known or suspected exposure to rodents (rats and mice) during the most recent year, as well as the preceding year.

### Descriptive analysis

The titre distributions for all six agents are presented crudely. For sv Bratislava and Icterohaemorrhagiae (with seroprevalence > 8%) titre distributions are also presented according to whether the horses were considered healthy or non-healthy. The numbers of horses tested and the seroprevalence to sv Bratislava and Icterohaemorrhagiae are presented crudely, by breed, age, gender, usage, region (host factors), season, pasture access, outdoor confinement and occurrence of rodents. Seroprevalences are presented for disease factors when at least 10 horses were affected.

### Multivariable analysis

Multivariable logistic regression was used (when seroprevalence was > 8%) to model two outcomes: titres ≥ 1:100 to the agents sv Bratislava and Icterohaemorrhagiae for associations with possible risk factors. For each outcome one "disease model" was constructed, with 1982 observations, deleting observations with missing information for either: breed, age, gender, racing (horses used for trotting competition and gallop racing were referred to in the multivariable analysis as "racing"), region or season. As risk factors in these models, breed, age, gender, racing, region, season, health status and all disease factors were considered (Table 2). For lameness it was decided to model only lameness any limb. For each outcome, one "pasture model" was further constructed where pasture access and outdoor confinement were also included as risk factors, containing 1763 observations (a number of observations missing in these variables) (Table 3). A third model was also tested: the "rodent model", where the rodent variable was simply forced on the final disease model (*n *= 1982).

**Table 2:** Multivariable results in the disease models for two different leptospira strains

		Bratislava				Icterohaemorrhagiae			
		
Variable	Category	est	se	OR	95% CI	est	se	OR	95%CI
Intercept		-3.814	0.309	-	-	-3.478	0.243	-	-
Age	(continuous)	0.049	0.016	1.0	1.0, 1.1	0.150	0.020	1.2	1.1,1.2
Gender	Mare	1.046	0.293	2.8	1.6, 5.1	NIM	-	-	-
	Gelding	0.687	0.295	2.0	1.1, 3.5	NIM	-	-	-
	Stallion	0	0	1.0	-	NIM	-	-	-
Breed	Coldblood	NIM	-	-	-	-1.026	0.452	0.4	0.1, 0.9
	Warmblood	NIM	-	-	-	-0.027	0.214	1.0	0.6, 1.5
	Thoroughbred	NIM	-	-	-	-0.462	0.409	0.6	0.3, 1.4
	Pony	NIM	-	-	-	-0.619	0.339	0.5	0.3, 1.0
	Standardbred	NIM	-	-	-	0	0	1.0	-
Racing	Not racing	0.982	0.164	2.7	1.9, 3.7	NIM	-	-	-
	Racing	0	0	1.0	-	NIM	-	-	-
Season	Jan-Mar	0	0	1.0	-	0	0	1.0	-
	Apr-June	0.722	0.185	2.1	1.4, 3.0	-0.234	0.261	0.8	0.5, 1.3
	July-Sept	0.295	0.195	1.3	0.9, 2.0	0.290	0.244	1.3	0.8, 2.2
	Oct-Dec	0.444	0.192	1.6	1.1, 2.3	0.581	0.235	1.8	1.1, 2.8
Health status	Healthy	NIM	-	-	-	-1.504	0.355	0.2	0.1, 0.4
	Non-healthy	NIM	-	-	-	0	0	1.0	-
Respiratory problems	Yes	0.572	0.263	1.8	1.1, 3.0	NIM	-	-	-
	No	0	0	1.0	-	NIM	-	-	-
Fatigue	Yes	1.279	0.351	3.6	1.8, 7.1	NIM	-	-	-
	No	0	0	1.0	-	NIM	-	-	-

The outcome is a titre of ≥ 1:100 to the respective agents. There were 1982 observations with 333 and 166 horses with positive titres to the respective agents

NIM – not in model, est – estimate, se – standard error, OR – odds ratio, CI – confidence interval

**Table 3:** Multivariable results in the pasture models for two different leptospira strains.

		Bratislava				Icterohaemorrhagiae			
		
Variable	Category	est	se	OR	95% CI	est	se	OR	95% CI
Intercept		-3.616	0.385	-	-	-3.449	0.260	-	-
Age, continuous		0.048	0.016	1.0	1.0, 1.1	0.149	0.019	1.2	1.1, 1.2
Gender	Mare	0.812	0.376	2.3	1.1, 4.7	NIM	-	-	-
	Gelding	0.679	0.378	2.0	0.9, 4.1	NIM	-	-	-
	Stallion	0	0	1.0	-	NIM	-	-	-
Racing	Not racing	1.045	0.183	2.8	2.0, 4.1	NIM	-	-	-
	Racing	0	0	1.0	-	NIM	-	-	-
Season	Jan-Mar	0	0	1.0	-	0	0	1.0	-
	Apr-June	0.802	0.198	2.2	1.5, 3.3	-0.349	0.283	0.7	0.4, 1.2
	July-Sept	0.340	0.209	1.4	0.9, 2.1	0.374	0.256	1.5	0.9, 2.4
	Oct-Dec	0.580	0.203	1.8	1.2, 2.7	0.652	0.246	1.9	1.2, 3.1
Paddock	Yes	-0.120	0.580	0.9	0.3, 2.8	NIM	-	-	-
	No	0	0	1.0	-	NIM	-	-	-
Riding in terrain	Yes	0.702	0.414	2.0	0.9, 4.5	NIM	-	-	-
	No	0	0	1.0	-	NIM	-	-	-
Pasture	None	NIM	-	-	-	0	0	1.0	-
	< 1 month	NIM	-	-	-	-1.010	0.380	0.4	0.2, 0.8
	1 < 3 months	NIM	-	-	-	-0.239	0.212	0.8	0.5, 1.2
	> 3 months	NIM	-	-	-	-0.965	0.260	0.4	0.2, 0.6
Interactions									
Paddock * Gender	Paddock, mare	0.296	0.164	1.3	1.0, 1.9	NIM	-	-	-
	Paddock, gelding	-0.490	0.620	0.6	0.2, 2.1	NIM	-	-	-
Riding in terrain * Racing	Riding in terrain, Not racing	-1.070	0.449	0.3	0.1, 0.8	NIM	-	-	-

The outcome is a titre of ≥ 1:100 to the respective agents. There were 1763 observations with 304 and 150 horses with positive titres to the respective agents.

NIM – not in model, est – estimate, se – standard error, OR – odds ratio, CI – confidence interval.

The same baselines were selected for host and pasture variables for both agents and all three models, selected on the basis of lowest seroprevalence for both outcomes. For the disease factors and outdoor confinement variables, the baselines were simply absence of this clinical sign/disease/procedure/type of outdoor confinement. Age, which was defined in whole years, was entered as a continuous variable, after verifying that age could be adequately considered linearly related to the outcome for the sv Bratislava and Icterohemorrhagiae models.

The reduction process was as follows. Variables that had a type 3 *p*-value < 0.2 remained in unreduced main effects models. All possible two-way interactions were tested, one at a time, upon the unreduced main effects models, and those that proved significant at *p *≤ 0.2 were retained for further reduction. A final *p*-value of 0.05 was considered significant.

The Hosmer-Lemeshow test based on percentiles was used for model validation. Over/under-dispersion was evaluated using the ratio of the deviance to the number of degrees of freedom. The SAS statistical software program (SAS Institute Inc., Cary, NC 27513, USA) was used to analyse the data, using the GENMOD procedure for logistic regression. Ninety-five percent confidence intervals (95% CI) for odds ratios (ORs) were constructed, taking exponentials of β_i _± 1.96 × SE.

## Results

### Seroprevalence

The highest seroprevalences for all investigated horses at a cut-off 1:100 were found for sv Bratislava (16.6%) and Icterohaemorrhagiae (8.3%). *Leptospira *seroprevalence and titre distribution for all investigated serovars for all horses are presented in Table 4. For sv Bratislava and Icterohaemorrhagiae, the seroprevalences for all horses by breed, age, gender, usage, region (host factors), season, pasture access, outdoor confinement and occurrence of rodents, are presented in Table 1. The distribution of seroprevalences for non-healthy horses (*n *= 1617) for sv Bratislava and Icterohaemorrhagiae by the disease factors asked are presented in Table 5.

**Table 4:** Number of serum samples from 2017 horses^a ^(healthy [*n *= 400] and non-healthy horses^b ^[*n *= 1617]) with titres to five *Leptospira *serovars obtained by microscopic agglutination test

	Number (%) of positive horses at each titre													
			
	Seropositive^c^		1:100		1:200		1:400		1:800		1:1600		1:3200	
Serovar	*n*	%	*n*	%	*n*	%	*n*	%	*n*	%	*n*	%	*n*	%
Bratislava	335	16.6	274	81.8	54	16.1	6	1.8	0	0	0	0	1	0.3
Healthy horses	65	16.3	49	75.4	14	21.5	2	3.1	0	0	0	0	0	0
Non-healthy horses	270	16.7	225	83.3	40	14.8	4	1.5	0	0	0	0	1	0.4
Icterohaemorrhagiae	168	8.3	101	60.1	41	24.4	16	9.5	7	4.2	2	1.2	1	0.6
Healthy horses	9	2.2	6	66.7	2	22.2	0	0	1	11.1	0	0	0	0
Non-healthy horses	159	9.8	95	59.7	39	24.5	16	10.1	6	3.8	2	1.3	1	0.6
Grippotyphosa	8	0.4	4	50.0	4	50.0	0	0	0	0	0	0	0	0
Pomona	11	0.5	8	72.7	0	0	3	27.3	0	0	0	0	0	0
Sejroe	24	1.2	15	62.5	5	20.8	3	12.5	0	0	0	0	1	4.2

^a^Horses were sampled during September 1997–98 from clinics associated with the Swedish Horserace Totalizator Board.

^b^Horses were grouped as healthy and non-healthy when the seroprevalence exceeded 8% at a cut-off 1:100. ^c^All horses with a titre ≥ 1:100

**Table 5:** Distribution of seroprevalences (%) for *Leptospira interrogans *serovars Bratislava and Icterohaemorrhagiae, cut-off 1:100, by the clinical/etiological categories/procedures asked for among non-healthy horses (*n *= 1617)^a^

		% positive horses	
		
Disease factor	No. of horses with clinical signs	sv Bratislava	sv Icterohaemorrhagiae
Sign, etiology or procedure^b^			
Behavioral problems	23	21.7	13.0
Colic	15	0	6.7
Fatigue	48	31.3	14.6
Fever	13	7.7	7.7
Inappetence	19	31.6	10.5
Integumentary problems	31	16.1	12.9
Lameness, any	905	17.2	10.5
Lame 1 limb	513	17.3	10.5
Lame ≥ 2 limbs	392	17.1	10.5
Radiographic examination^c^	24	8.3	4.2
Respiratory signs	113	20.4	9.7
Staggering	10	20.0	0
Stiffness	49	22.4	8.2
Tendon injuries	70	17.1	12.9
Unwillingness to be ridden	49	24.5	10.2
Wasting	24	20.8	4.2

^a^Horses sampled during September 1997–98 from clinics associated with the Swedish Horserace Totalizator Board. ^b^Abortion, ophthalmological diseases and diarrhea were excluded from the clinical signs, etiological categories and/or procedures because < 10 horses were said to have been affected. Cases of septic arthritis were excluded from the joint problems. ^c^Admitted for various problems to the equine clinics by veterinarians in horse practice.

### Multivariable analysis

#### The disease models

(Table 2) contained 1982 observations with 333 and 166 horses with titres positive to the respective agents: sv Bratislava and Icterohaemorrhagiae. In the sv Bratislava model, colic was omitted and in the sv Icterohaemorhagiae model, the interaction between breed and season was omitted, both because of collinearity. The ratio of deviance to degrees of freedom was 0.82 in the sv Bratislava model and 0.52 in the sv Icterohaemorrhagiae model, the latter indicating under-dispersion. The *p*-values from the Hosmer-Lemeshow tests were 0.54 and 0.20, indicating well-fitting models. The rodent variable was not significant in any model when included on the reduced disease models.

#### The pasture models

(Table 3) contained 1763 observations with 304 and 150 horses with titres positive to the respective agents: sv Bratislava and Icterohaemorrhagiae. In these models the ratios of deviance to degrees of freedom were 0.84 and 0.53 for the respective agents, the ratio for the sv Icterohaemorrhagiae model indicated under-dispersion. The *p*-values from the Hosmer-Lemeshow tests were 0.98 and < 0.001, indicating that only the sv Bratislava model fitted the data well. In the sv Icterohaemorrhagiae model, the largest positive deviance residuals approached 3, and were found for young horses and horses sampled in April – June.

For **sv Bratislava **the disease model included three host factors (age, gender, racing), season and health status (Table 2). The pasture model included the same factors and also "paddock" and "riding in terrain", but not health status (Table 3). In both the disease and pasture models it was found that seroprevalence increased with age and that the seasons April – June and October – December had higher seroprevalences than other seasons. With respect to genders, mares and geldings had higher odds ratios (ORs) than stallions in the disease model. However, there was an interaction with paddock in the pasture model, indicating that mares in paddocks had a slightly higher seroprevalence than geldings in paddocks. The main effect of "non-racing" was similar in both models (ORs 2.8 and 2.7). However, "non-racing" interacted with "riding in terrain" in the pasture model, with a low OR for individuals having both these attributes (0.3). "Respiratory problem" yielded an OR of 1.8 and "fatigue" was associated with an OR of 3.6 with respect to seropositivity in the disease model.

The **sv Icterohaemorrhagiae **disease model included two host factors (breed, age), season and health status (Table 2). The pasture model included one host factor (age), plus season and pasture (Table 3). In both disease and pasture models, seropositivity increased with age and the seroprevalence was higher in October – December than for other seasons. Only the disease model contained "breed", where coldbloods and ponies had lower levels of seropositivity. In the disease model, healthy horses also had a lower OR (0.2).

## Discussion

### Study design

These serum samples were previously used to study the seroprevalence to *Borrelia burgdorferi sensu lato *and granulocytic *Ehrlichia *spp. (*Anaplasma phagocytophilum*) [28]. Several issues in that paper are relevant also for the present study. For example, the population including both healthy and diseased horses is not a cross-section of the Swedish horse population. More horses with some kind of problems are seen than in the base population and competition horses as well as active sport horses are probably overrepresented, while foals, brood-mares and stallions used for breeding are probably underrepresented. However, the stratified and multivariable approaches compensate for this problem to some degree, making possible estimation of odds ratios (ORs) and percentages relevant to subgroups. We cannot say whether the clinical signs preceded the titres or vice versa. Seropositivity is not a pretext for an ongoing disease but a measure of exposure. In our cross-sectional study, we don't know if the horses with titres developed clinical signs later. One further problem was the relatively large number of missing values for the questions concerning pasture access. Also, we believe that the answers to the rodent exposure questions might not have accurately reflected the rodent situation on the premises. This was because the questioned person was probably often not the person best acquainted with rodent density and further there might also be differences regarding interpretation of rodent density.

During the investigation, two different strains of sv Bratislava were used as antigens, the reference strain Jez and a laboratory strain of unknown origin. We included the latter as it had been used earlier to investigate samples in the routine diagnostics at our laboratory and there had then been a tendency to "high" seroprevalence with this strain (unpublished observations) and sometimes difficulties in interpreting the results. It was demonstrated that the overall seroprevalence for the investigated horses was considerably higher for the laboratory strain (34.8%) than for the reference strain Jez (16.6%), Ellis *et al*. [3] also found differing seroprevalences for horses investigated with different antigens of sv Bratislava. However, the laboratory strain was not included in this study as it was not a reference strain. We conclude that use of different strains as antigen for a serovar can give differing seroprevalences. For correct and meaningful comparisons between studies it is therefore important to use the same reference strain as antigen, also suggested by Ellis *et al*. [3].

### Seroprevalence, host factors, pasture and outdoor confinement factors

The seroprevalence for the horses in this study was highest for sv Bratislava. Also, in other investigations the seroprevalence was higher for sv Bratislava than for other serovars and the horse has been suggested to be a maintenance host for this serovar [3,16,20,29]. For both sv Bratislava and Icterohaemorrhagiae, seropositivity increased with age. The same has also been reported by others [20,21,29,30]. It is likely that horses, during their life-span, encounter leptospires from the environment or from other horses and titres can persist for a long time.

With respect to gender, mares and geldings had, compared with stallions, a higher OR of seropositivity to sv Bratislava in the disease model. Also, horses not used for racing had a higher OR of seropositivity to sv Bratislava in both models. One interpretation could be that stallions and racing horses are more often kept individually and may therefore be less exposed to shedding from other horses. Management may differ too. In a study by Lees and Gale [30], it was shown that track horses that were managed individually had lower ORs than rodeo horses that were managed in groups, for all investigated serovars including sv Bratislava and Icterohaemorrhagiae. In our study we did not ask whether horses were kept individually or in groups.

Breed was significant in the disease model, where interestingly ponies and coldbloods had a lower OR of seropositivity to sv Icterohaemorrhagiae. One explanation could be that in the summer, grazing horses do not come into contact with other kinds of feed or material that may be contaminated by urine from small rodents. Maybe some breeds, e.g. coldblood and ponies, usually have greater access to pasture than other breeds and therefore are less exposed to rodents in the stable in the summer. Also, management factors can differ between breeds. For sv Icterohaemorrhagiae the maintenance host is the rat, which serves a as reservoir of infection [1]. In this study, there was no correlation between exposure to rodents and seroprevalence for *Leptospira *and in the multivariable models rodents were removed for both investigated serovars.

In the study by Barwick *et al*. [31], soil and water index were the only factors associated with the risk of exposure to all investigated serovars. In Europe, the hedgehog is known as a wildlife host of sv Bratislava [32]. In this study, we did not take into account the variation in wildlife or access to stagnant water, e.g. in ponds. In the pasture model for sv Bratislava, there was an interaction between "riding in terrain" and the "racing" variables. Combining the estimates in this interaction and using racing horses not ridden (trained) in terrain as the baseline (OR = 1), all other categories had OR point estimates ≥ 2. The highest point estimate was found for non racing horses not ridden in terrain (OR = 2.8), the findings from this interaction being difficult to explain. A hypothesis may be that horses living close to cities are ridden in the terrain less and may become more exposed to shedding from other horses due to kept on smaller areas per horse than horses out in the country.

Furthermore, in the pasture model, there was an interaction between mares and paddocks (yard without grass). Judging from the combined estimates using stallions with no paddock as baseline, indicated that mares that were kept in paddocks (exp^(0.812-0.120+0.296) ^= OR 2.7) had a higher seroprevalence than geldings in paddocks (OR = 1.1), the gender difference being smaller when both these genders did not specifically have access to paddocks (data not shown). This interaction too is difficult to explain.

### Season

The present study indicates seasonal variations in leptospiral titres in both models. For sv Icterohaemorrhagie, horses had the highest seroprevalence in October – December. Small rodents living in the wild usually invade stables and houses in late summer and autumn, which can give an increased infection incidence during this period. For sv Bratislava the highest seroprevalence was found in April – June and also October – December. Moisture, moderately warm temperature and neutral or mildly stagnant water are essential for survival of the organism in the environment [1]. Probably most horses get infected during the warmer season, in spring and summer. The separate peak in October-December may be explained by that horses are usually regrouped in the beginning of the autumn when taken from pasture to paddocks. They may then be exposed to shedding from new horses when they infect each other. Seasonal variation has also been reported for other animal species in other countries e.g. pigs in Vietnam [33].

### Disease factors

In the disease model, serum antibody titres to sv Bratislava and Icterohaemorrhagie were not generally associated with the disease factors included in this study, with two exceptions: horses with fatigue and also horses with respiratory problems had higher levels of seropositivity to sv Bratislava. In a study by Van der Ingh *et al*. [16], fever and respiratory distress were observed in foals with leptospirosis and it was suggested that leptospiral infection was spread by direct contact between the horses in the outbreak. Moreover, it was demonstrated for sv Icterohaemorrhagiae that healthy horses had lower levels of seropositivity than non-healthy horses. The explanation may be that factors that promote equine health also protect against infection of *Leptospira *spp, which is why healthy horses are less susceptible. In Sweden, the clinical disease of leptospirosis in horses is seldom diagnosed though serological reactions – especially to sv Bratislava and also Icterohaemorrhagiae – are sometimes found.

## Conclusion

From a clinical point of view, this study showed that there was no significant association between clinical signs and disease and positive titres to sv Bratislava or Icterohaemorrhagiae (except for the association between respiratory problems and fatigue and seropositivity to sv Bratislava). The results of this study suggest that horses with increasing age and exposed to factors associated with outdoor life had an increased seroprevalence for sv Bratislava, indicating that horses get infected from outdoor and/or are exposed to shedding from other horses i.e. related to management. For sv Icterohaemorrhagiae, management possibly plays a role as ponies and coldbloods as well as healthy horses had lower seroprevalence. Overall, the age of the horse should be taken into consideration when evaluating the titre as the average healthy horse has a higher titre than a young horse.

## Competing interests

The authors declare that they have no competing interests.

## Authors' contributions

VB is the main author of the paper. All authors (VB, AE, PF, AG, EOE) contributed to the design of the study. PF was in charge of collecting all clinical information and blood sampling. VB was responsible for the laboratory investigations. AE conducted the statistical analysis of this paper. VB participated in the statistical analysis. VB drafted the manuscript in collaboration with AE. All authors read and approved the final manuscript.
